# *Microphallus pseudopygmaeus* (Digenea) infects phylogenetically distant gastropods, with signs of host-linked genetic divergence

**DOI:** 10.1051/parasite/2026016

**Published:** 2026-03-30

**Authors:** Alina I. Sokolova, Kirill V. Galaktionov, Anna Gonchar

**Affiliations:** 1 Department of Invertebrate Zoology, Saint Petersburg State University Saint Petersburg Russia; 2 Laboratory of Parasitic Worms and Protists, Zoological Institute of the Russian Academy of Sciences, Universitetskaya emb. 1 Saint Petersburg 199034 Russia

**Keywords:** Host specificity, Host switching, First intermediate host, Intraspecific divergence, Speciation

## Abstract

Host-switching between distantly related host species offers rare insight into how parasites overcome compatibility barriers and initiate evolutionary divergence. *Microphallus pseudopygmaeus* is exceptional among digeneans in its ability to infect gastropods from two distantly related subclasses, Vetigastropoda and Caenogastropoda. This study aimed to test the hypothesis about the species status of *M. pseudopygmaeus* and clarify its host range. We obtained partial sequences of the *cox1* gene, 28S rDNA and ITS2 for *M. pseudopygmaeus* from nine host species, including *Margarites* spp. (Vetigastropoda). The data on the conservative and variable markers, phylogenetic and barcoding gap analyses, supported the unity of the species and its broad specificity to the first intermediate hosts. The *cox1*-based haplotype network revealed host-associated genetic divergence, particularly in isolates from *Margarites* spp. and *Cryptonatica affinis*. This pattern may result from localized circulation of the parasite in the regions where certain host species, such as *Margarites* spp., dominate in the absence of periwinkles, creating ecological conditions that could promote reproductive isolation and incipient speciation. This work opens up the prospects of using *M. pseudopygmaeus* as a model for studying host-switching and speciation in parasites.

## Abstract (fr)

Le passage d’un hôte à un autre entre des espèces hôtes éloignées offre un aperçu rare de la façon dont les parasites surmontent les barrières de compatibilité et initient une divergence évolutive. *Microphallus pseudopygmaeus* est exceptionnel parmi les digènes par sa capacité à infecter des gastéropodes appartenant à deux sous-classes éloignées, les Vetigastropoda et les Caenogastropoda. Cette étude visait à tester l’hypothèse concernant le statut d’espèce de *M. pseudopygmaeus* et à préciser son spectre d’hôtes. Nous avons obtenu des séquences partielles du gène *cox1*, de l’ADNr 28S et de l’ITS2 pour *M. pseudopygmaeus* chez neuf espèces d’hôtes, dont *Margarites* spp. (Vetigastropoda). Les données sur les marqueurs conservés et variables, l’analyse phylogénétique et l’analyse du *barcode gap*, ont confirmé l’unité de l’espèce et sa large spécificité envers les premiers hôtes intermédiaires. L’analyse du réseau d’haplotypes basé sur le gène *cox1* a révélé une divergence génétique liée à l’hôte, notamment chez les isolats provenant de *Margarites* spp. et de *Cryptonatica affinis*. Ce schéma pourrait résulter d’une circulation localisée du parasite dans les régions où certaines espèces hôtes, comme *Margarites* spp., dominent en l’absence de littorines, créant ainsi des conditions écologiques susceptibles de favoriser l’isolement reproductif et une spéciation naissante. Ces travaux ouvrent la voie à l’utilisation de *M. pseudopygmaeus* comme modèle d’étude du changement d’hôte et de la spéciation chez les parasites.

## Introduction

Parasites pose several challenging questions in evolutionary biology, to name just a few – the origins of parasitism, the rationale for life cycle complexity, the nature of virulence dynamics, the force balancing specificity and generalism. The question of how parasites may retain specificity while rapidly responding to changes is intriguing, and it is currently considered that the primary mode of parasite evolution is not co-speciation but rather host-switching [14, 40]. The latter must be particularly frequent in parasites featuring multi-host life cycles, perfectly exemplified by digeneans.

In the life cycles of these parasitic flatworms, parthenogenetic (asexual) generations (sporocysts and/or rediae) develop in the first intermediate hosts, metacercariae in the environment or in the second intermediate hosts, and maritae (sexual adults) in the definitive hosts. Colonization of new hosts has been the main driver of digenean evolution [5, 8, 18, 32]. Switching definitive hosts can be a hallmark of new lineages, *e.g.* family Brachycladiidae (fishes to cetaceans, [22]); a major divergence event within lineages, *e.g.*, in Schistosomatidae (birds to mammals, [8]); or a repeated event contributing to a wide host range, *e.g.*, in Cyathocotylidae [1]. Switching second intermediate hosts is also one of the pathways for digeneans to diversify and reach definitive hosts with varying feeding preferences (*e.g.*, in Opecoelidae, [59], Hemiuridae [51]). Evident history of switching first intermediate hosts by digeneans [5, 8, 58, 80] goes along with their remarkable specificity at the fine taxonomic level.

One digenean species is usually restricted to one molluscan genus (*e.g.*, most schistosomes [8], *Opisthorchis felineus* [61], *Podocotyle atomon* [50]), or even species (*e.g.*, *Himasthla elongata* [35], *Catatropis onobae* [37], *Neophasis annarichae* [47], *Orthosplanchnus arcticus* [46, 49]). In some cases, the range of suitable first intermediate hosts is wider and includes multiple members of the same family [23] or even superfamily [52]. Such cases of broader specificity can provide insights into how the colonization of new taxa of first intermediate hosts can drive speciation in digeneans. As a model for this type of study, we propose the species *Microphallus pseudopygmaeus* Galaktionov, 1980 (Digenea: Microphallidae).

**Figure 1:**
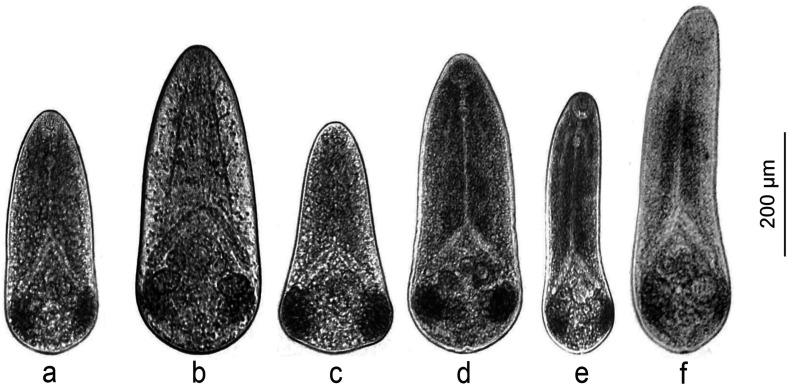
Metacercariae of microphallids of the “pygmaeus” group, microphotographs. (a) *M. pseudopygmaeus*, (b) *M. pygmaeus*, (c) *M. triangulatus*, (d) *M. kurilensis*, (e) *M. piriformes*, (f) *M. calidris*.

**Figure 2:**
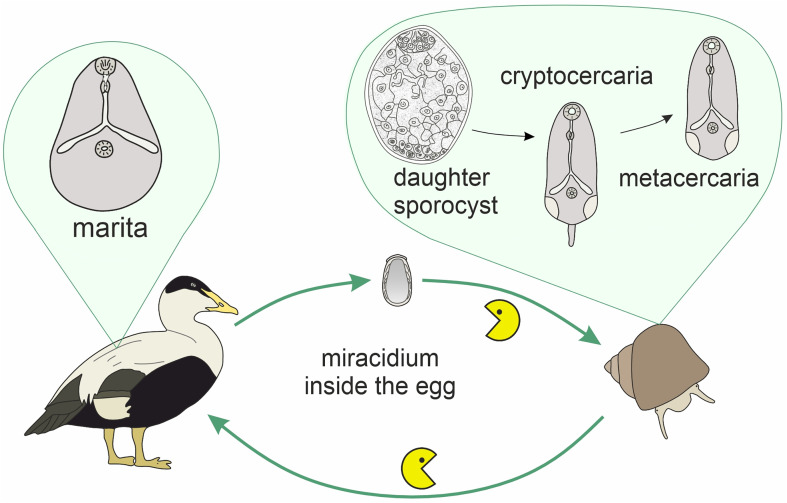
Life cycle of microphallids of the “pygmaeus” group. Definitive hosts: marine birds; first intermediate hosts: marine gastropods (typically, periwinkles); trophic transmission: the Pac-Man icon.

*Microphallus pseudopygmaeus* belongs to a group of closely related species referred to as the “pygmaeus” group [31]. These digeneans have no free-living cercariae, and metacercariae (Fig. 1) develop directly within daughter sporocysts in the first intermediate host. The life cycle therefore includes only the definitive hosts (marine anatids, mainly eiders; or gulls) and the first intermediate hosts (shared by all members of the “pygmaeus” group are periwinkles, *Littorina* spp.) (Fig. 2). However, *M. pseudopygmaeus* has expanded its range of first intermediate hosts to as many as 17 gastropod species, some of which are phylogenetically distant. This conclusion was initially based on morphological observations [25, 28, 29] and later supported by sequencing of the ITS1, ITS2 and 28S rDNA regions [31]. To fully appreciate the host range of *M. pseudopygmaeus*, we have now expanded the dataset to include the widest available diversity of hosts, and the variable mtDNA marker. The results inspired us to discuss biogeography, host switching and speciation, and envision further studies.

## Material and methods

### Sampling

**Figure 3:**
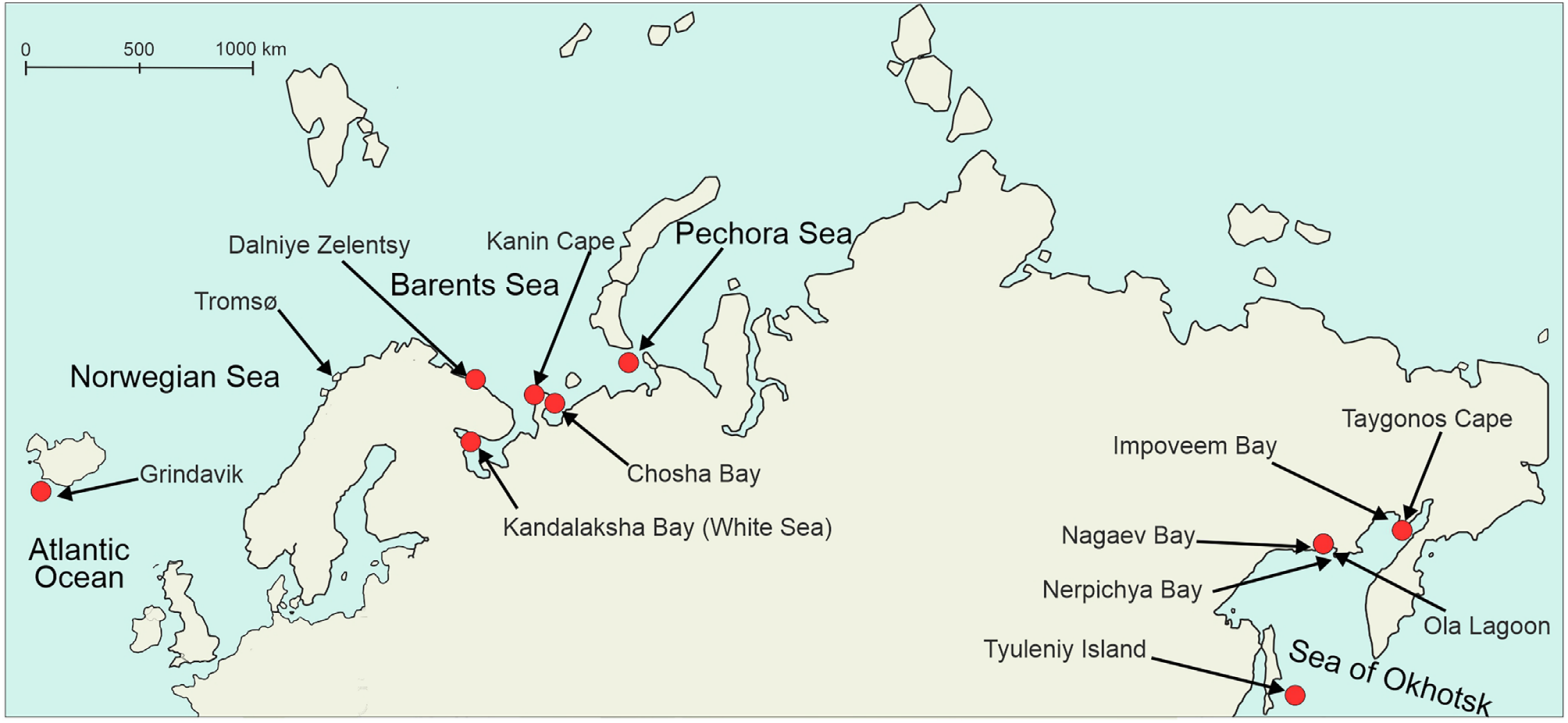
Map of all sampling sites (indicated by arrows) and *M. pseudopygmaeus* sampling sites (red dots). Closely spaced localities (*e.g.*, within the Pechora Sea and the White Sea) are not shown on the map.

Sampling took place on the shores of the northern European seas (Norwegian, Barents, White, Pechora) and the Sea of Okhotsk (Table 1, Fig. 3) in 2003–2025. In addition, we used 10 isolates of microphallids collected by Georgii Kremnev and Darya Krupenko in 2023–2024. All the samples were from the gastropod first intermediate hosts (Table 2). Three periwinkle species belonging to the “saxatilis” complex (*Littorina saxatilis*, *Littorina arcana* and *Littorina compressa*) co-occur at our Barents Sea sampling location [38]. They may be difficult to distinguish when the snail is castrated due to trematode infection. In these cases, we listed them as belonging to the “saxatilis” species complex. During low tide, we collected littoral snails, while the mollusks *Cryptonatica affinis* (Gmelin, 1791) and *Margarites* spp. were gathered from the sublittoral zone by dredging and diving. We kept mollusks in containers filled with sea water, and then dissected them to detect infection. Microphallid sporocysts and metacercariae were identified and preserved in 96% ethanol. In addition to *M. pseudopygmaeus*, we sampled other members of the “pygmaeus” group (*Microphallus pygmaeus* (Levinsen, 1881) Baer, 1944 [55], *Microphallus piriformes* Galaktionov, 1983 [26], *M. pseudopygmaeus*, *Microphallus triangulatus* Galaktionov, 1984 [27], *Microphallus calidris* Belopolskaja and Ryzhikov [4], *Microphallus kurilensis* Galaktionov, Regel and Atrashkevich, 2010 [34]; Fig. 1) and *Microphallus similis* (Jägerskiöld, 1900) Baer, 1944 [42].

**Table 1:** Geographical locations of sampling sites.

Region	Site	Coordinates
White Sea (Kandalaksha Bay)	Levin Navolok Bay	66°17′ 51″ N 33° 27′ 36″ E
	Korovya Varakka Cliff	66°18′34.5″ N 33°37′13.2″ E
	Yakovleva Bay	66°18′49.9″ N 33°50′24.6″ E
	Cape Kindo	66°32′19.5″ N 33°11′44.7″ E
Barents Sea	Dalniye Zelentsy	69°07′07.0″ N 36°02′07.0″ E
	Chosha Bay	67°47′41.0″ N 46°29′29.0″ E
	Kanin Cape	68°39′36.0″ N 43°25′24.0″ E
Norwegian Sea	Tromsø	69°40′58.0″ N 18°56′34.0″ E
Pechora Sea	Dolgiy Island	69°16′28.0″ N 59°05′32.0″ E
	Vaygach Island	70°25′03.4″ N 58°47′50.0″ E
	Malyi Zincovyi Island	69°50′21.0″ N 59°29′30.0″ E
	Gubistyi Island	69°49′33.6″ N 59°25′40.5″ E
Sea of Okhotsk	Nagaev Bay	59°32′26.0″ N 150°40′48.0″ E
	Tyuleniy Island	48°30′02.5″ N 144°38′59.9″ E
	Taygonos Cape	60°43′36.7″ N 160°24′06.9″ E
	Impoveem Bay	61°17′37.0″ N 159°55′16.8″ E
	Ola Lagoon	59°34′46.2″ N 151°17′42.2″ E
	Nerpichya Bay	59°17′34.0″ N 152°08′00.0″ E
Atlantic Ocean (SW Iceland)	Grindavik	63°49′49.3″ N 22°25′54.7″ W

**Table 2:** Material used in this study and the corresponding GenBank accession numbers.

*ID*/Museum voucher	Host species	Geographic origin		GenBank accession numbers		
		Region	Site	*cox1*	28S rDNA	5.8S rDNA– ITS2
***M. pseudopygmaeus***						
Mps02/2025.11.12.001	*Littorina saxatilis* (Olivi, 1792)	PS	Dolgiy Island	PQ611031		
Mps03/2025.11.12.002	*L. saxatilis*	PS	Dolgiy Island	PQ611032		
Mps04/2025.11.12.003	*L. saxatilis*	BS	Dalniye Zelentsy	PQ611033		
Mps06/2025.11.12.004	*Littorina sitkana*	SO	Taygonos Cape	PQ611034		
	R. A. Philippi, 1846					
Mps07/2025.11.12.005	*L. saxatilis*	PS	Vaygach Island	PQ611035		
Mps08/2025.11.12.006	*L. saxatilis*	PS	Vaygach Island	PQ611036		
Mps09/2025.11.12.007	*L. saxatilis*	PS	Vaygach Island	PQ611037		
Mps10/2025.11.12.008	*L. saxatilis*	PS	Vaygach Island	PQ611038		
Mps12/2025.11.12.009	*Littorina obtusata* (Linnaeus, 1758)	BS	Dalniye Zelentsy	PQ611039		
Mps16/2025.11.12.010	*Margarites helicinus* (Phipps, 1774)	BS	Dalniye Zelentsy	PQ611040	PQ836125	PV789575
Mps21/2025.11.12.011	*L. saxatilis*	PS	Dolgiy Island	PQ611041		
Mps24/2025.11.12.012	*L. saxatilis*	PS	Dolgiy Island	PQ611042		
Mps25/2025.11.12.013	*L. saxatilis*	PS	Vaygach Island	PQ611043		
Mps27/2025.11.12.014	*L. saxatilis*	PS	Malyi Zincovyi Island	PQ611044		
Mps29/2025.11.12.015	*L. saxatilis*	BS	Malyi Zincovyi Island	PQ611045		
Mps31/2025.11.12.016	*L. saxatilis*	PS	Malyi Zincovyi Island	PQ611046		
Mps32/2025.11.12.017	*L. saxatilis*	PS	Malyi Zincovyi Island	PQ611047		
Mps35/2025.11.12.018	*L. saxatilis*	PS	Malyi Zincovyi Island	PQ611048		
Mps39/2025.11.12.019	*L. saxatilis*	PS	Malyi Zincovyi Island	PQ611049	PQ836126	PQ836137
Mps42/2025.11.12.020	*L. saxatilis*	PS	Malyi Zincovyi Island	PQ611050		
Mps44/2025.11.12.021	*Lacuna vincta* (Montagu, 1803)	PS	Gubistyi Island	PQ611051	PQ836127	PQ836136
Mps45/2025.11.12.022	*L. vincta*	PS	Gubistyi Island	PQ611052		
Mps46/2025.11.12.023	*L. vincta*	PS	Gubistyi Island	PQ611053		
Mps47/2025.11.12.024	*L. vincta*	PS	Gubistyi Island	PQ611054		
Mps48/2025.11.12.025	*L. vincta*	PS	Gubistyi Island	PQ611055		
Mps54/2025.11.12.026	*Onoba aculeus* (Gould, 1841)	WS	Levin Navolok Bay	PQ611056	PQ836128	PQ836135
Mps57/2025.11.12.027	*O. aculeus*	WS	Levin Navolok Bay	PQ611057		
Mps58/2025.11.12.028	*O. aculeus*	WS	Levin Navolok Bay	PQ611058		
Mps64/2025.11.12.029	*C. affinis*	BS	Dalniye Zelentsy	PQ611059	PQ836129	PQ836134
Mps66/2025.11.12.030	*L. vincta*	BS	Dalniye Zelentsy	PQ611060		
Mps67/2025.11.12.031	*M. helicinus*	BS	Dalniye Zelentsy	PQ611061	PV789548	PV789576
Mps68/2025.11.12.032	*M. helicinus*	BS	Dalniye Zelentsy	PQ611062	PV789549	PV789577
Mps69/2025.11.12.033	*M. helicinus*	BS	Dalniye Zelentsy	PQ611063		PV789578
Mps70/2025.11.12.034	*L. saxatilis*	WS	Levin Navolok Bay	PQ611064		
Mps71/2025.11.12.035	*L. obtusata*	WS	Levin Navolok Bay	PQ611065	PQ836130	PQ836133
Mps72/2025.11.12.036	*L. saxatilis*	WS	Levin Navolok Bay	PQ611066		
Mps73/2025.11.12.037	*L. obtusata*	WS	Levin Navolok Bay	PQ611067		
Mps74/2025.11.12.038	*L. saxatilis*	WS	Levin Navolok Bay	PQ611068		
Mps75/2025.11.12.039	*L. saxatilis*	WS	Levin Navolok Bay	PQ611069		
Mps76/2025.11.12.040	*L. saxatilis*	WS	Levin Navolok Bay	PQ611070		
Mps77/2025.11.12.041	*L. saxatilis*	WS	Levin Navolok Bay	PQ611071		
Mps78/2025.11.12.042	*L. saxatilis*	WS	Levin Navolok Bay	PQ611072		
Mps79/2025.11.12.043	*L. saxatilis*	WS	Levin Navolok Bay	PQ611073		
Mps86/2025.11.12.044	*L. sitkana*	SO	Tyuleniy Island	PQ611074		
Mps87/2025.11.12.045	*L. sitkana*	SO	Nagaev Bay	PQ611075		
Mps95/2025.11.12.046	*M. helicinus*	WS	Korovya Varakka Cliff	PQ611076		PV789579
Mps100/2025.11.12.047	*O. aculeus*	BS	Dalniye Zelentsy	PQ611077		
Mps103/2025.11.12.048	Littorina arcana*	BS	Dalniye Zelentsy	PQ611078		
	Hannaford-Ellis, 1978					
Mps105/2025.11.12.049	*M. groenlandicus* (Gmelin, 1791)	WS		PQ611079		
Mps106/2025.11.12.050	*M. groenlandicus*	WS		PQ611080		
Mps107/2025.11.12.051	*C. affinis*	BS	Dalniye Zelentsy	PQ611081	PV789550	PQ836132
Mps112/2025.11.12.052	*L. obtusata*	BS	Kanin Cape	PQ611082		
Mps117/2025.11.12.053	*L. obtusata*	BS	Kanin Cape	PQ611083		
Mps96/2025.11.12.054	*O. aculeus*	AO	Grindavik	PX560772		
Mps110/2025.11.12.055	*L. saxatilis*	BS	Chosha Bay	PX560776		
Mps111/2025.11.12.056	*L. saxatilis*	BS	Chosha Bay	PX560777		
Mps119/2025.11.12.057	*M. groenlandicus*	WS			PV789551	PV789580
Mps120/2025.11.12.058	*M. groenlandicus*	WS		PX560778	PV789552	PV789581
Mps98/2025.11.12.059	*O. aculeus*	BS	Dalniye Zelentsy	PX560773		
Mps101/2025.11.12.060	*O. aculeus*	BS	Dalniye Zelentsy	PX560774		
Mps102/2025.11.12.061	*O. aculeus*	BS	Dalniye Zelentsy	PX560775		
2.AC/2025.11.12.062	*M. groenlandicus*	WS	Cape Kindo	PX560768		
3.AC/2025.11.12.063	*M. groenlandicus*	WS	Cape Kindo	PX560769		
12.AC/2025.11.12.064	*L. saxatilis*	WS		PX560770		
23.AA/2025.11.12.065	*M. groenlandicus*	WS	Cape Kindo	PX560771		
						
***M. pygmaeus***						
Mps17/2025.11.12.066	*L. saxatilis*	BS	Dalniye Zelentsy	PV418132		
Mps23/2025.11.12.067	*L. saxatilis*	PS	Vaygach Island	PV418133		
Mps28/2025.11.12.068	*L. saxatilis*	PS	Malyi Zincovyi Island	PV418134		
Mps62/2025.11.12.069	*L. obtusata*	BS	Dalniye Zelentsy	PV418135		
Mpyg1/2025.11.12.070	*L. saxatilis*	PS	Vaygach Island	PV418128		
Mpyg2/2025.11.12.071	*L. saxatilis*	PS	Vaygach Island	PV418130		
Mpyg30/2025.11.12.072	*L. saxatilis*	BS	Dalniye Zelentsy	PV418129		
Mpyg31/2025.11.12.073	*L. saxatilis*	BS	Dalniye Zelentsy	PV418131		
***M. triangulatus***						
Mtr1/2025.11.12.074	*L. saxatilis*	WS	Levin Navolok Bay	PV418151		
Mtr2/2025.11.12.075	*L. saxatilis*	PS	Malyi Zincovyi Island	PV418152		
***M. kurilensis***						
Mkur1/2025.11.12.076	*L. sitkana*	SO	Nerpichya Bay	PV418114		
Mkur5/2025.11.12.077	*L. sitkana*	SO	Nerpichya Bay	PV418116		
Mcal2/2025.11.12.078	*Larus schistisagus*	SO	Impoveem Bay	PV418115		
	Stejneger, 1884					
***M. calidris***						
Mcal1/2025.11.12.079	*L. sitkana*	SO	Impoveem Bay	PQ900221		
***M. piriformes***						
Mpir3/2025.11.12.080	*L. saxatilis* *	BS	Dalniye Zelentsy	PV418117		
Mpir4/2025.11.12.081	*L. saxatilis* *	BS	Dalniye Zelentsy	PV418118		
Mpir19/2025.11.12.082	*L. saxatilis*	BS	Chosha Bay	PV418119		
Mpir20/2025.11.12.083	*L. saxatilis*	BS	Chosha Bay	PV418120		
Mpir21/2025.11.12.084	*L. saxatilis*	BS	Chosha Bay	PV418121		
Mpir22/2025.11.12.085	*L. saxatilis*	BS	Chosha Bay	PV418122		
Mpir23/2025.11.12.086	*L. saxatilis*	BS	Chosha Bay	PV418123		
Mpir24/2025.11.12.087	*L. obtusata*	BS	Chosha Bay	PV418124		
Mpir25/2025.11.12.088	*L. saxatilis*	BS	Kanin Cape	PV418125		
Mpir26/2025.11.12.089	*L. saxatilis*	BS	Chosha Bay	PV418126		
Mpir30/2025.11.12.090	*L. obtusata*	WS	Levin Navolok Bay	PV418127		
***M. similis***						
Msm1/2025.11.12.100	*L. sitkana*	SO	Ola Lagoon	PV418136		
Msm2/2025.11.12.101	*L. sitkana*	SO	Ola Lagoon	PV418137		
Msm4/2025.11.12.102	*L. saxatilis*	NS	Tromsø	PV418138		
Msm5/2025.11.12.103	*L. obtusata*	AO	Grindavik	PV418139		
Msm6/2025.11.12.104	*L. sitkana*	SO	Ola Lagoon	PV418140		
Msm7/2025.11.12.105	*L. sitkana*	SO	Ola Lagoon	PV418141		
Msm8/2025.11.12.106	*L. sitkana*	SO	Ola Lagoon	PV418142		
Msm9/2025.11.12.107	*L. sitkana*	SO	Ola Lagoon	PV418143		
Msm12/2025.11.12.108	*L. saxatilis*	NS	Tromsø	PV418144		
Msm13/2025.11.12.109	*L. obtusata*	NS	Tromsø	PV418145		
Msm17/2025.11.12.110	*L. sitkana*	SO		PV418146		
Msm21/2025.11.12.111	*Littorina littorea* (Linnaeus, 1758)	BS	Dalniye Zelentsy	PV418147		
Msm23/2025.11.12.112	*L. sitkana*	SO	Nagaev Bay	PV418148		
Msm24/2025.11.12.113	*L. sitkana*	SO	Nagaev Bay	PV418149		
Msm25/2025.11.12.114	*L. sitkana*	SO	Nagaev Bay	PV418150		

Mollusks marked * belong to the “saxatilis” species complex, but were not further identified to the species level. Regions: PS – Pechora Sea; BS – Barents Sea; WS – White Sea; SO – Sea of Okhotsk; NS – Norwegian Sea; AO – Atlantic Ocean (SW Iceland).

To identify microphallids of the “pygmaeus” group, we prepared wet mounts as previously described [25, 69, 71]. Slides with metacercariae in a drop of distilled water were heated for *ca.* 1 min at 70 °C using a heating table. Then, completely relaxed and flattened metacercariae were identified based on morphological characters, and the reference photographs were made using an Olympus CH40 compound microscope (Olympus Optical Co. Ltd., Tokyo, Japan) equipped with an Olympus XC-30 digital camera (Olympus Optical Co. Ltd.) (Fig. 1). We did not use other morphological methods because comprehensive data about the structure of *M. pseudopygmaeus* metacercariae from different snail hosts are already available [28, 29], and here we focused on identifying intra- and interspecific genetic variability.

The material was preserved in 96% ethanol for further studies. We deposited voucher tissue samples (paragenophores) to the Trematoda voucher collection of the Zoological Institute of the Russian Academy of Science (ZISP) under accession numbers 2025.11.12.001–2025.11.12.114; their correspondence to the GenBank accession numbers of the sequences is provided in Table 2.

Nucleotide sequence data reported in this paper are available in the GenBank database under the accession numbers PQ611031–PQ611083, PQ728074–PQ728079, PQ836125–PQ836130, PQ836132–PQ836137, PV789575–PV789581 and PX560768–PX560778.

### DNA extraction, PCR and sequencing

We removed a single sporocyst from ethanol, placed it in a new 1.5-mL microtube, and let any remaining ethanol evaporate by keeping a tube open for 1–3 min. To extract DNA, we added 200 μL of the 5% ion exchange resin Chelex 100 (Bio-Rad, Hercules, CA, USA) and 2 μL of proteinase K (Evrogen, Moscow, Russia) to each tube, then incubated samples at 56 °C for 4 hours while shaking at 850 rpm on a thermomixer (BioSan, Riga, Latvia). To inactivate the proteinase, the samples were then boiled at 90 °C for 8 min. After that, we centrifuged the tubes for 10 min at 16,874 ×*g* (Eppendorf 5418). The DNA in the supernatant was transferred to a new tube and stored at −20 °C.

To amplify the partial *cox1* gene, 5.8S–ITS2 region and D1–D3 domains of 28S rRNA gene, we used the primers listed in Table 3. The primer JB3 was modified based on the draft mitogenome assembly of *M*. *pseudopygmaeus* (unpublished data by K.V. Galaktionov) to enhance PCR outcome.

**Table 3:** PCR primers used in this study.

Markers	Primers	Sequences of forward (F) and reverse (R) primers (5′–3′)	Reference
28S rRNA	digl2	F, AAGCATATCACTAAGCGG	[76]
	1500R	R, GCTATCCTGAGGGAAACTTCG	
5.8S–ITS2	3S	F, CGGTGGATCACTCGGCTCGTG	[6]
	ITS2.2	R, CCTGGTTAGTTTCTTTTCCTCCGC	[17]
*cox1*	JB3	F, TTTTTTGGGCATCCTGAGGTTTAT	[7]
	JB3-mps	F, TT**C**TT**C**GG**T**CATCC**A**GAGGTTTAT	Our modification
	trem.cox1.rrnl	R, AATCATGATGCAAAAGGTA	[45]

Amplification was performed in 20 μL reaction mixtures containing 2 μL DNA template, 0.5 μL forward and reverse primer each (10 pmol/μL), 4 μL ScreenMix-HS reaction mix (Evrogen, Russia), and 13 μL Super-Q water.

PCR conditions for the *cox1* fragment were as following: initial denaturation at 95 °C for 5 min, 35 cycles (95 °C – 30 s, 48 °C – 30 s, 72 °C – 1 min), and final elongation at 72 °C for 10 min (T100 BioRad). To amplify the fragment of 5.8S–ITS2, we used the protocol from [47]. To amplify the D1–D3 domains of the 28S rDNA, we used the touchdown PCR protocol with a sequential decrease in the annealing temperature of 55.5 °C (10 cycles) – 55 °C (10 cycles) – 54.5 °C (15 cycles). PCR-products were size-separated by electrophoresis in a 1% agarose gel (SE-1, Helicon), stained with ethidium bromide and photographed in the UV light using ChemiDoc BioRad.

Sanger sequencing (forward and reverse) was performed on an automated capillary sequencer ABI 3500xl (Applied Biosystems, Foster City, CA, USA) at the Centre for Molecular and Cell Technologies, St Petersburg University.

### Data processing and analysis

Sequence data were processed in Geneious Prime 23.2.1 [43]. To estimate genetic distances, we used Mega 11 [75]. For new rDNA sequences and those publicly available from GenBank, we calculated mean intra- and interspecific distances with the maximum likelihood estimation method [75]. For all specimens of the “pygmaeus” group microphallids and *M. similis*, we calculated pairwise p-distances in the *cox1* fragment, and used these data to build a histogram of distance frequencies in R [65] and RStudio IDE version 2024.12.0 [62] with a ggplot2 package [81].

Species delimitation hypotheses were tested based on *cox1* sequence data using assemble species by automatic partitioning (ASAP) [63] implemented in the iTaxoTools toolkit, with simple distances and default parameters [77]. For the *cox1*-based phylogenetic reconstruction, we used *M. similis* as an outgroup and microphallids of the “pygmaeus” group as an ingroup; identical sequences were removed from the alignment. Bayesian inference (BI) was performed in MrBayes, v. 3.2.7a [70] with GTR + inv + G model for 1,000,000 generations (sampling and diagnosing frequencies 1,000, 25% burnout), making sure the runs converged. For the maximum likelihood approach (ML), we used RAxML-NG v. 1.2.2 [44] implemented in raxmlGUI 2.0.13 [20] with the integrated model selection (HKY + FO + I+G4m), “ML + transfer bootstrap expectation + consensus” option, one run, and 100 replicates. The BI tree was visualized, and the poorly supported nodes (posterior probabilities below 0.75) were collapsed into polytomies. For nodes that were also recovered in the ML analysis, both BI and ML support values were printed. ASAP scores were visualized and color-coded next to the resulting tree.

We also created a *cox1*-haplotype network in PopART 1.7 with the TCS algorithm [13].

For species in the “pygmaeus” group, we also searched for the robust diagnostic nucleotide combinations: combinations of nucleotides at specified sites of the alignment, unique for a particular species and sufficient to differentiate it from all other taxa in a dataset. This search was performed in MolD [21] implemented in iTaxoTools [77].

We prepared the figures in CorelDRAW Graphics Suite, v. 24.0.0.301 [16].

## Results

### Nuclear markers

We obtained eleven 28S rDNA and thirteen 5.8S–ITS2 rDNA sequences for isolates of *M. pseudopygmaeus* from different molluscan hosts: *L. saxatilis*, *L. obtusata*, *Lacuna vincta*, *O. aculeus*, *C. affinis*, *M. helicinus*, and *M. groenlandicus.* These data were analyzed together with the sequences of *M. pseudopygmaeu*s and six other *Microphallus* species available in GenBank (HM584122–HM584142, HM584175–HM584199, [31]; MG783583–MG783584, MG783588–MG783589, [30]; AY220625, [76]).

The trimmed 28S rDNA alignment was 971 bp long and included 29 sequences. There were no intraspecific variations except for one *M. piriformes* sequence differing by one substitution. The distances between the species of the “pygmaeus” group (the number of base substitutions per site) were 0–0.034 (Supplementary Table S1). The trimmed 5.8S–ITS2 rDNA alignment was 266 bp long and included 36 sequences. There were also no intraspecific variations except for two sequences of *M. pseudopygmaeus* from *C. affinis* which differed by one substitution. The distances between the species of the “pygmaeus” group were 0.06–0.027 (Supplementary Table S2).

### Mitochondrial marker *cox1*

We obtained 104 new *cox1* sequences, their length ranging from 795 to 963 bp (*M. pseudopygmaeus* – 64, *M. pygmaeus* – 8, *M. triangulatus* – 2, *M. kurilensis* – 3, *M. piriformes* – 11, *M. calidris* – 1, *M. similis* – 15). The reverse primer trem.cox1.rrnl flanks a region outside the *cox1* gene; this is a non-coding fragment directly adjacent to the *cox1* CDS, followed by tRNA. To be on the safe side, for GenBank we annotated the *cox1* CDS only and left the remaining fragment unannotated.

#### Species delimitation

**Figure 4:**
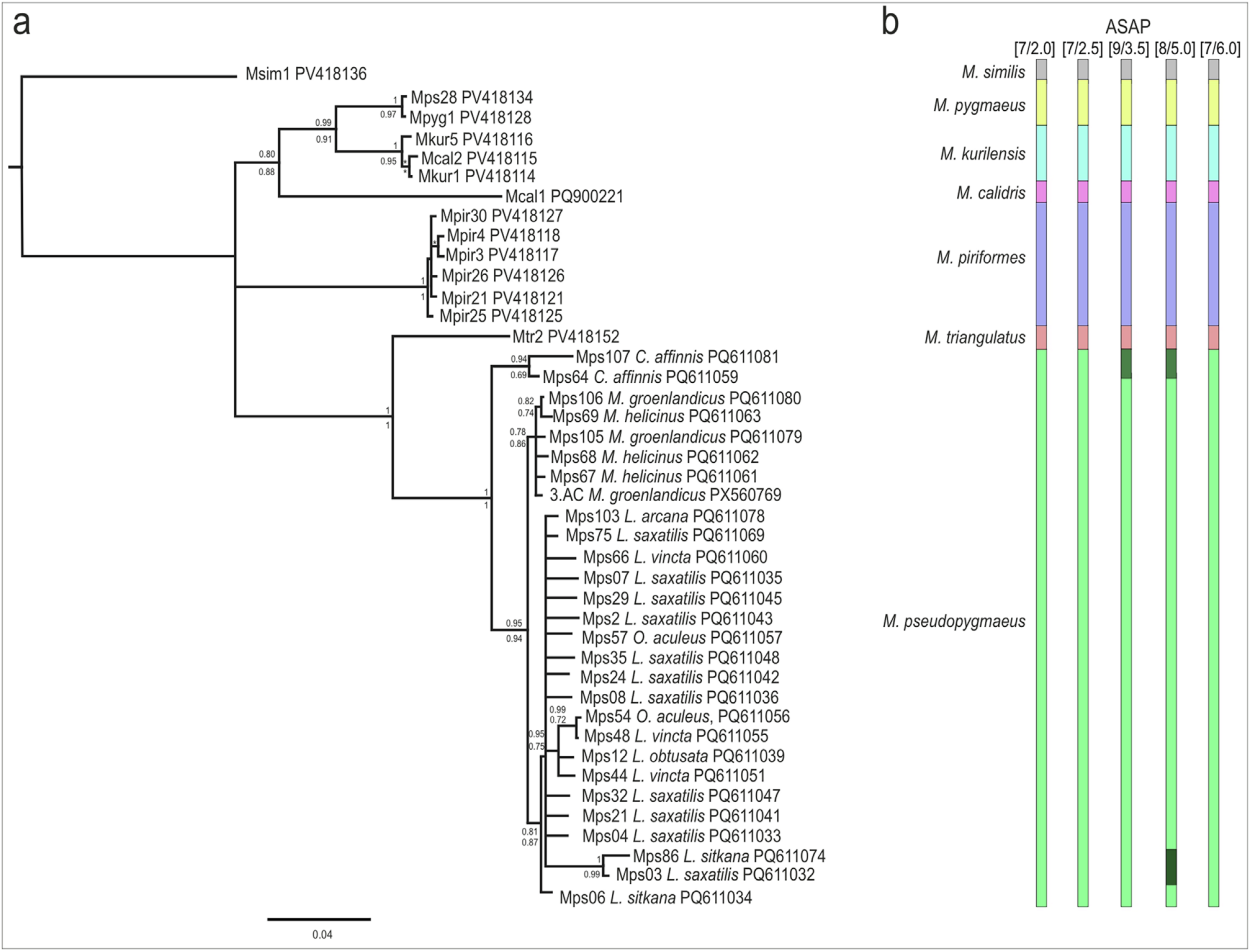
Phylogenetic relationships of the “pygmaeus” group microphallids based on *cox1* sequences. (a) Bayesian phylogenetic tree. Poorly supported nodes (posterior probability < 0.75) were collapsed into polytomies. For nodes also recovered in the maximum likelihood (ML) analysis, both BI and ML support values are shown. (b) Five best ASAP scores and partition numbers (in brackets); and color-coded putative species boundaries corresponding to the clades in the tree.

In the *cox1*-based phylogeny (801 bp alignment including 42 sequences), each microphallid of the “pygmaeus” group was recovered as a well-supported clade (Fig. 4a). This matches well the results of the *cox1*-based ASAP analysis (801 bp alignment including 104 sequences): the minimum ASAP score (2.0), and thus the highest probability, was assigned to the partition including seven subsets (Fig. 4b). These subsets correspond to the seven assumed species in the alignment, supporting the hypothesis that *M. pseudopygmaeus* is a single species.

**Figure 5:**
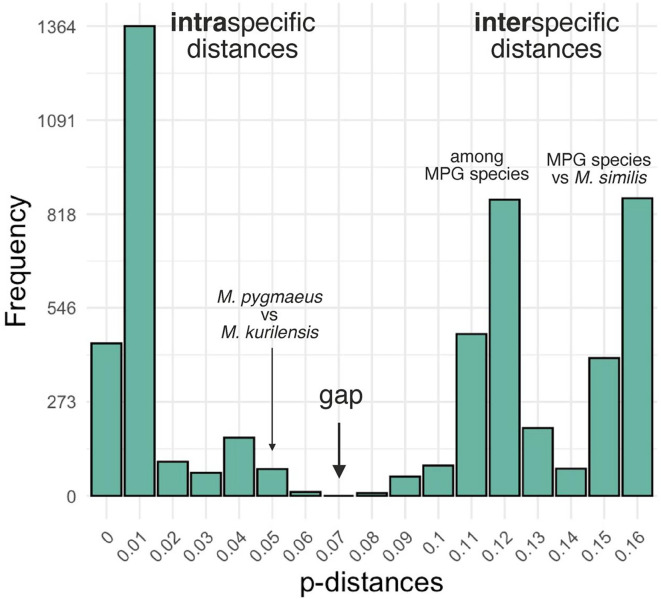
Frequency distribution of pairwise genetic distances in the *cox1* gene fragment (803 bp) for seven species of *Microphallus*. MPG, microphallids of the “pygmaeus” group.

The boundary value of p-distances was 0.0631, and the barcoding gap is visualized in Figure 5. Most of the pairwise p-distances within *M. pseudopygmaeus* ranged from 0.00 to 0.014, with five outlier specimens displaying distances above this range, up to 0.056. Intraspecific distances among microphallids of the “pygmaeus” group were above 0.079, and mostly above 0.010. However, there was one exception: *M. pygmaeus* and *M. kurilensis* specimens differed by just 0.048–0.051; despite distances below the threshold value, these species are still consistently recognized as distinct in the ASAP analysis (Fig. 4b). Distances between *M. similis* and the “pygmaeus” group specimens were 0.140–0.156. The complete *cox1* distance matrix is provided in Supplementary Table S3; the alignment used for this matrix and ASAP analysis is in Supplementary File.

Diagnostic nucleotide combinations (DNCs) were identified in MolD for all members of the “pygmaeus” group microphallids, based on the 801 bp *cox1* alignment as used in ASAP analysis, and were as follows: C188 and A191 in *M. pygmaeus*; C143 and T191 in *M. kurilensis*; C2 and A29 in *M. calidris*; C3, T8 and G188 in *M. piriformes*; C8 and C65 in *M. triangulatus*; and C188 and A191 in *M. pseudopygmaeus*. When the analysis was run for the *M. pseudopygmaeus* isolates subdivided by host (*Cryptonatica affinis*, *Margarites* spp. and all other), DNCs were only recovered for isolates from *C. affinis*: A 35, T38, and C80.

#### Haplotype network

The alignment contained 74 *cox1* sequences: 64 for *M. pseudopygmaeus* from nine species of molluscan hosts and five each for *M. pygmaeus* and *M. similis* to exemplify interspecific distances. The total length of the alignment was 800 bp.

We discovered 28 haplotypes among the putative *M. pseudopygmaeus* samples. Most of the haplotypes differed by one substitution. There was a minimum of 83 substitutions between *M. pseudopygmaeus* and the closely related species *M. pygmaeus*; and 109 between *M. pseudopygmaeus* and *M. similis*.

**Figure 6:**
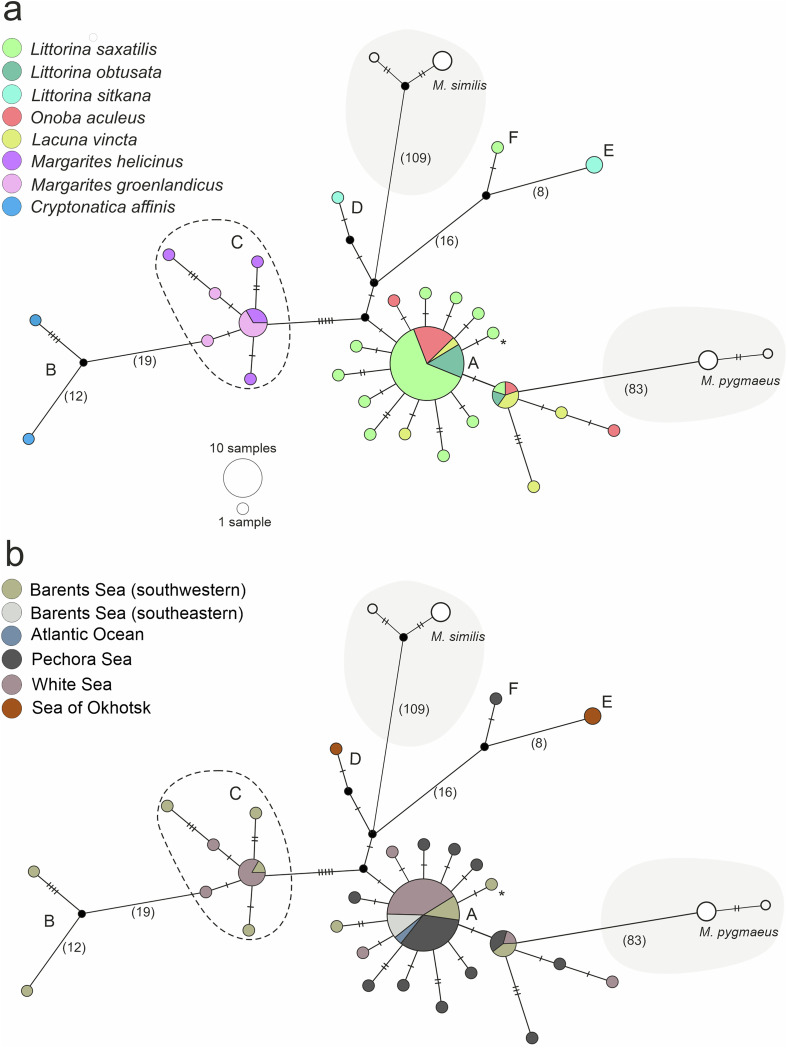
Haplotype networks for *M. pseudopygmaeus*, also including several samples of *M. pygmaeus* and *M. similis*, based on the fragment of the *cox1* gene (800 bp). Circle sizes represent haplotype frequencies, hatch marks represent substitutions (if more than five, replaced with numbers). The asterisk marks the sample from *L. arcana* (belongs to “saxatilis” species complex). Haplotypes are colored according to parasite host species (a) and sampling location (b). The sampling locations in the Barents Sea: Dalniye Zelentsy (southwestern), Chosha Bay, Kanin Cape (southeastern); in the Pechora Sea: Dolgiy Island, Vaygach Island, Malyi Zincovyi Island, Gubistyi Island; in the White Sea: Levin Navolok Bay, Cape Kindo, Korovya Varakka Cliff; in the Sea of Okhotsk: Tyuleniy Island, Taygonos Cape, Nagaev Bay; in the Atlantic Ocean: Grindavik.

The distribution of *M. pseudopygmaeus* haplotypes among the species of molluscan hosts is illustrated with colors in Figure 6a. The dominant haplotype A included 27 samples from *L. saxatilis*, *L. obtusata*, *Lacuna vincta* and *Onoba aculeus*. Two samples of *M. pseudopygmaeus* from *Cryptonatica affinis* had the most divergent haplotypes and were only moderately similar to each other (B); they differed from the dominant haplotype A by 30–38 substitutions. Samples of *M. pseudopygmaeu*s from *Margarites* spp. (*M. helicinus* and *M. groenlandicus*) grouped together (haplogroup C); they differed from the dominant haplotype A by 6–10 mutations. Sequences of the samples from *L. sitkana* formed two haplotypes which differed markedly from each other: one (D) was close to the dominant haplotype (differs by four mutations), while the second one (E) stood alone (differed by 25 substitutions from the dominant haplotype A).

The distribution of *M. pseudopygmaeus* haplotypes among the sampling regions is illustrated with colors in Figure 6b. The genetically distinct haplotype E included the geographically distinct specimens from the North Pacific (Sea of Okhotsk). However, one specimen from this region appeared separately as haplotype D. The dominant haplotype A was shared by specimens originating from all the sampled regions in the North Atlantic. However, one specimen from the North Atlantic (haplotype F, Pechora Sea) appeared relatively closer to haplotype E of North Pacific origin.

## Discussion

*Microphallus pseudopygmaeus* has been described as a species able to infect an extremely wide range of molluscan first intermediate hosts [24, 29]. Later, its host range was partially confirmed with rDNA sequence data [31]. However, considering the occurrence of cryptic and pseudocryptic species among trematodes, the integrity of *M. pseudopygmaeus* required revision with a variable genetic marker. In this study, we focused on the *cox1* gene data and analyzed additional evidence that *M. pseudopygmaeus* is indeed a single species.

### Species delimitation in the microphallids of the “pygmaeus” group

Results of the phylogenetic and ASAP analyses match the previous assumption that “pygmaeus” group comprises six species: *M. pygmaeus*, *M. piriformes*, *M. pseudopygmaeus*, *M. triangulatus*, *M. calidris* and *M. kurilensis*. This implies the unity of *M. pseudopygmaeus*, with all its specimens from various first intermediate host species. Phylogenetic relationships suggest that it is a sister species to *M. triangulatus* (Fig. 4), similarly to the previous rDNA-based findings [31]. There is no doubt that *M. pseudopygmaeus* and *M. triangulatus* are distinct species, considering genetic (Supplementary Table S3) and morphological (Fig. 1) differences. The distinction between the sister species *M. pygmaeus* and *M. kurilensis* is less obvious: while both are recognized in the ASAP analysis, intraspecific distance is below the overall threshold (Fig. 5). However, the ASAP algorithm is more sophisticated than simple distance comparison, and together with other evidence [31], our *cox1* data support that these are two species.

Within *M. pseudopygmaeus*, there are two genetically diverged subgroups that may be recognized as independent partitions with the less-robust ASAP scores (3.5; 5.0) (Fig. 4). One corresponds to haplotypes D, E and F in the haplotype network (Fig. 6) and is likely intraspecific and geography-driven (see below). The second cluster corresponds to specimens from *C. affinis*; it is further discussed in section “Naticoidea (Caenogastropoda, Littorinimorpha)”. As for all the other *M. pseudopygmaeus* specimens, their integrity does not seem to be questioned by the *cox1* data.

Below, we first summarize all data on the range of first intermediate hosts used by *M. pseudopygmaeus*, then put these data in the broader digenean context, discuss the likely evolutionary patterns in *M. pseudopygmaeus* and consider the relevance of our results for evolutionary parasitology.

### Gastropod first intermediate hosts of *M. pseudopygmaeus*: an overview

#### Littorinoidea (Caenogastropoda, Littorinimorpha)

Periwinkles are the original and the most abundant intertidal first intermediate hosts of *M. pseudopygmaeus* [31]. Our dataset includes samples from *L. obtusata*, *L. saxatilis*, *L. arcana* (all Atlantic) and *L. sitkana* (Pacific) and from one more littorinid *Lacuna vincta*. Patterns of genetic variation among these samples seem to be driven by geography rather than the host species: diverged haplotype E from *L. sitkana* has North Pacific origin (Fig. 6b). This is further discussed in section “Biogeographic history”.

*Littorina scutulata* A. Gould, 1849 was also reported to be used by *M. pseudopygmaeus* as a host [25]. Although adults resulting from the experimental infection of mice with metacercariae isolated from *L. scutulata* were initially identified as *M. pygmaeus* [11], revision of the whole mounts clarified that the worms actually were *M. pseudopygmaeus* [24].

The species *M. pseudopygmaeus* has not been found in the common periwinkles *Littorina littorea* (Linnaeus, 1758). Apropos, *M. pygmaeus* does infect this mollusk, with prevalence as high as 16.7% on the White Sea (unpublished data by D. Fedorov). Neither *M. pseudopygmaeus* nor other “pygmaeus” microphallids have been found in *Littorina squalida* Broderip and G. B. Sowerby I, 1829, the Far East sister species of *L. littorea* (KG, personal observations).

#### Rissooidea (Caenogastropoda, Littorinimorpha)

Previously, the experimental infections of the common eider with the microphallid metacercariae from *O. aculeus* produced the maritae identified as *M. pseudopygmaeus* [29], and the specimen from *O. aculeus* was identical in 28S rDNA sequence with the specimen of *M. pseudopygmaeus* from *L. saxatilis* [31]. Our *cox1* data go in line with these findings.

Additionally, *M. pseudopygmaeus* was reported from another species of Rissooidea, *Boreocingula martyni*, in the Sea of Okhotsk [31], but we have no molecular evidence to verify this.

#### Naticoidea (Caenogastropoda, Littorinimorpha)

Metacercariae from the moonsnails *C*. *affinis* have previously been identified as *M. pseudopygmaeus*, despite certain morphological differences [28, 29]. We now found genetically that they are also relatively diverged from the other North Atlantic specimens, with one substitution in ITS2, distant *cox1* haplotypes that form a clade on the tree and share a diagnostic combination of three nucleotides. Still, for now we consider the hypothesis that *C*. *affinis* is indeed one of the hosts of *M. pseudopygmaeus* more plausible. This may be a dynamic situation which should be further investigated.

Prevalence of *M. pseudopygmaeus* infection in *C. affinis* varies substantially. On Kolguyev Island, Pechora Sea, prevalence is as high as 11.1–25% [29, 33]. In the White and Barents Seas, among 297 *C. affinis* snails dissected in 2018–2021 [48] only one (0.3%) was found to be infected with *M. pseudopygmaeus*.

#### Truncatelloidea (Caenogastropoda, Littorinimorpha)

It is molecularly confirmed that *M. pseudopygmaeus* infects one species from the superfamily Truncatelloidea, *Falsicingula athera* Bartsch, 1967 [31], but *Peringia ulvae* (Pennant, 1777) and *Ecrobia ventrosa* (Montagu, 1803) (also Truncatelloidea) had never been found infected on the White Sea, with 94,758 and 31,956 specimens examined, respectively [54]. There is a sporadic report of *M. pseudopygmaeus* infection in *E. ventrosa* from Skerjafjörður, Iceland [74]. This report could indicate the ongoing colonization of new hosts by *M. pseudopygmaeus*.

#### Trochoidea (Vetigastropoda, Trochida)

*Microphallus pseudopygmaeus* has been known to infect snails belonging to two genera of Trochoidea: *Solariella* (Solariellidae) and *Margarites* (Margaritidae). The latter hosts have now been confirmed with molecular data: at least we found no evidence to challenge this fact. Our isolates from *M. helicinus* (five) and *M. groenlandicus* (six) grouped in a haplotype network (Fig. 6a). This may reflect certain specialization to the molluscs (Vetigastropoda) that are phylogenetically most distant from the other first intermediate hosts of this parasite (Caenogastropoda), which is further discussed in the section “Host-associated divergence”.

### Evolutionary pathways in *M. pseudopygmaeus*

#### Biogeographic history

The ancestral first intermediate hosts of the “pygmaeus” microphallids were *Littorina* spp. which formed in the North Pacific and spread to the Atlantic during the Pliocene opening of the Bering Strait [9], along with their parasites [31]. The glaciation at the end of the Pliocene resulted in the rise of the Bering land bridge (“Beringia”), leading to allopatric speciation in both periwinkles [66, 68] and their microphallids in the Atlantic [31]. Indeed, most species from the “pygmaeus” group infecting only periwinkles occur either in the Atlantic (*M. pygmaeus*, *M. piriformes*) or in the Pacific (*M. calidris*, *M. kurilensis*).

Occurrence of *M. pseudopygmaeus* across Eurasia, and likely gene flow between the North Atlantic and North Pacific, is possible because this species has extended the range of its first intermediate host species. The primary role of molluscan hosts is reinforced by the short (8–9 days) lifespan of *M. pseudopygmaeus* maritae [29], making the long-distance transfer of this parasite within the bird definitive host alone impossible. Arctic waterfowl migrate along Eurasian and North American coastlines, and could potentially facilitate such transfer. However, without a continuous distribution of suitable molluscan hosts along these extensive migration routes, their role in connecting parasite populations remains limited. Instead, successful transfer requires a series of localities where both hosts occur and can support the life cycle.

The common eider, a key definitive host of *M. pseudopygmaeus*, has circumpolar distribution [78], and so do some of its first intermediate hosts, such as *Margarites* spp., *Solariella* spp. and *C. affinis*. Eiders routinely forage for these molluscs at depths of up to 20 m. Consequently, the life cycle of *M. pseudopygmaeus* can be completed throughout the Arctic coastal regions, enabling the trans-Arctic dispersal of the parasite. This is in contrast to other species of the “pygmaeus” group microphallids, where the only first intermediate hosts are periwinkles which are absent between the Kara Gates and the Chukchi Sea [66, 67, 72, 73] and cannot support “stepwise” connection between the Pacific and Atlantic populations of parasites.

Given the limited material available from the North Pacific, ideas on the relationships between the North Pacific and the North Atlantic *M. pseudopygmaeus* are preliminary. Assuming the North Pacific origin of the species [31], the samples from the Sea of Okhotsk would represent an ancestral lineage. These samples include two divergent haplotypes, D and E. A plausible, though tentative, interpretation is that the more distinct haplotype E is closer to the ancestral state, while haplotype D, which is relatively similar to the European variants, might reflect a secondary re-entry into the Pacific from the Atlantic during one of the warm Pleistocene interglacials. Likewise, the occurrence of haplotype F in the North Atlantic could result from a relatively recent colonization event, compared to haplotype A. These hypotheses are now speculative and may change with the accumulation of new data.

#### Host switching

In principle, host-switching requires that the encounter and compatibility filters [14] open. Encounter filter can open when geographical and/or ecological barriers between the potential hosts are affected by environmental perturbations. In the case of *M. pseudopygmaeus*, this could have happened during the glaciation cycles in late Pliocene–Pleistocene, when both actual and potential hosts concentrated in the glacial refugia [38, 41]. Compatibility filter can open when a parasite adopts a mechanism to deal with defensive systems of potential new hosts [39]. In the case of *M. pseudopygmaeus*, this must have been quite massive (considering the host range), but not universal, because some common gastropods, *e.g.* Buccinidae, and even the periwinkles of the subgenus *Littorina (L. littorea* and *L. squalida*) were not colonized.

*Microphallus pseudopygmaeus* is a good illustration of Digenea in general, where, despite strict specificity to the first intermediate hosts, the major mode of evolution was not co-speciation but host-switching [5, 8, 18, 32]. Colonization of new first intermediate hosts occurred through resource tracking [5]. Among the best-studied examples is host-switching from Planorbidae (Heterobranchia) to Potamiomsidae (Caenogastropoda) in the *Schistosoma sinensium* lineage, following the uplift of the Tibetan Plateau [3, 53, 57, 79]. Brooks and McLennan [10] suggest two models of resource tracing: phylogenetic tracking (a suitable resource is restricted to the host clade) and sequential colonization (a suitable resource is plesiomorphically or convergently widely spread among the potential hosts). The species *M. pseudopygmaeus* probably follows the second model: many coastal molluscs can serve as first intermediate host for it, but colonization is limited by a compatibility filter.

#### Host-associated divergence

Within *M. pseudopygmaeus*, all the currently available isolates from *Margarites* spp. grouped in the *cox1* haplotype network, suggesting certain intraspecific specialization. This may be attributed to selective success of snail infection, a step towards sympatric speciation by assortative survival [15]. An important question is how it is supported if different molluscan hosts of *M. pseudopygmaeus* occur in sympatry.

Perhaps specialization could be facilitated in those Arctic areas where periwinkles are absent while *Margarites* spp. are common, and so are the marine anatids. Rapid egg production by *M. pseudopygmaeus* maritae (within 2–4 days [24, 29]) allows this parasite to circulate locally in such areas, for example, in Franz-Joseph Land [36]. This assumption is also consistent with lack of divergence in *M. pseudopygmaeus* from *O. aculeus* and *L. vincta* (their distribution ranges are virtually the same as that of *Littorina* spp. [72]). Distinctness of *M. pseudopygmaeus* from *C. affinis* is consistent, too, because these mollusks are also Boreal-Arctic. However, with just two samples, there is not much room for discussion. For both *Margarites* spp. and *C. affinis*, it is important to highlight that the specimens in our dataset were collected in the same locations as *Littorina*, *Lacuna* and *Onoba*. *Margarites* spp. and *L. vincta* even share the very same microhabitats in the kelp forests.

There are examples of intraspecific genetic differentiation in digeneans guided by the second intermediate or definitive hosts [2, 60], and it can readily be explained by host use preferences or ecological specialization. However, genetic structure corresponding to the first intermediate host species has never before been documented. However, a similar situation has recently been described in the cestode *Ligula intestinalis* (Linnaeus, 1758) Gmelin, 1790 [56], showing genetic differentiation among the different fish second intermediate host species in sympatry, while also sharing a bird definitive host [60]. The authors believe reproductive isolation may be established despite the continued gene flow, and hypothesize incipient speciation through disruptive selection. In *M. pseudopygmaeus*, can we estimate whether speciation is ongoing?

#### Possible speciation

Though the hypothesis remains speculative, it is worth discussing that host-associated divergence in *M. pseudopygmaeus* may be a step towards speciation.

The life cycle of *M. pseudopygmaeus* involves passive transmission only: the eggs get accidentally ingested by the snails and the snails get ingested by the birds, mostly eiders. So, no behavioural features of a parasite may have contributed to host switching and the potential speciation. Instead, *M. pseudopygmaeus* miracidium, mother and daughter sporocysts have probably gained characters necessary to successfully establish infection in the new snail hosts. Parthenogenetic reproduction of these successful mutants would result in hundreds (and as many as 3,000) metacercariae sharing this genotype, enhancing the spread of the newly emerged form.

Next, we would expect selection against the intermediate forms – those poor at infecting both the original and the new hosts. Disruptive selection is plausible because we detected divergence in the most unrelated hosts, *Margarites* spp., which are more likely to bear drastic differences in the defence mechanisms, intestinal environment, haemocoel composition, *etc.* If the other hosts share more of these features, they can be tackled by the genetically similar parasites, not yielding any specialization. The species *C. affinis* is also quite distinct in its ecology (shell-boring predators, not grazers) and geographical distribution, and samples from this host are genetically diverged. Due to limited sample size for *C. affinis*, discussion below focuses mostly on *Margarites* spp.

For the reproductive isolation to endure, assortative survival in the first intermediate hosts must be followed by assortative mating in the definitive hosts. On the one hand, the eiders’ diet includes all the *M. pseudopygmaeus* first intermediate hosts. Mating between maritae originating from different gastropod hosts seems unavoidable at first, and this would contribute to the constant genetic mixing in a parasite. On the other, there are plausible mechanisms that could support consistent mating among maritae tracing the same molluscan origin. First, the *M. pseudopygmaeus* maritae are very short-lived and also tend to pass through the bird’s intestine in a compact yet numerous group originating from the same infected snail, likely mating within this group. Moreover, selfing may be suspected in extremely small *M. pseudopygmaeus* maritae (around 300 μm long) that invariably contain embryonated eggs in natural infections (KG, unpublished results). The probability of selfing in digeneans is not clear, and in microphallids specifically it has been tested by culturing individual worms *in vitro*, with both positive [12] and negative [19, 64] results.

Distinct local transmission hotspots in high latitudes (discussed section “Naticoidea (Caenogastropoda, Littorinimorpha)”) and in Pleistocene glacial refugia could also contribute to the possibility of speciation. The definitive hosts, common eiders, concentrate in coastal areas of islands for breeding, and in Franz Josef Land infected *Margarites* spp. occur precisely in the vicinity of these colonies [36]. Such hotspots are likely to be partially isolated from one another due to the short lifespan of the maritae. This isolation may favor mutation accumulation in local parasite populations associated with local first intermediate hosts.

## Conclusion

Taken together, our data confirm the exceptionally wide range of gastropods that serve as first intermediate hosts for the digenean *M. pseudopygmaeus*. This species also demonstrates the genetic divergence associated with its vetigastropod hosts, *Margarites* spp., and most probably with its naticid host *C. affinis*. This makes it an excellent host-parasite system to explore evolution of host specificity, host switching, and speciation. This model could be meaningful even beyond the parasite discourse because specialization within *M. pseudopygmaeus* is likely reinforced in the Arctic regions, and in circumstances comparable to those that occurred during the Pleistocene glacial cycles when many modern species formed.

## Supplementary material

*Supplementary Table S1*: The average pairwise genetic distances in 28S rDNA fragment (971 bp). *Supplementary Table S2*: The average pairwise genetic distances in ITS2–5.8S rDNA fragment (266 bp). *Supplementary Table S3*: p-distances for sequences from specimens of Microphallus of the „pygmaeus“ group and M. similis. *Supplementary File:* ASAP_samples_104_801. The supplementary material of this article is available at https://www.parasite-journal.org/10.1051/parasite/2026016/olm.
